# Does new infrastructure improve public health? Evidence from smart city pilot program in China

**DOI:** 10.3389/fpubh.2025.1655119

**Published:** 2025-09-11

**Authors:** Sha Zhou, Jia Ren

**Affiliations:** ^1^School of Physical Education, Hunan First Normal University, Changsha, China; ^2^School of Economics and Finance, Shanghai International Studies University, Shanghai, China

**Keywords:** smart city, new infrastructure, digital, public health, China

## Abstract

As a key pillar of new infrastructure development, smart city construction seeks to meet residents’ growing demand for high quality urban living by creating environments that are technologically integrated. To identify the causal effect of new infrastructure development on public health, we exploit the staggered launch of the national smart city pilot program in China as a quasi-natural experiment. We apply a multi-period difference-in-differences approach with 18,993 individual-level observations from 2010 to 2020. Our empirical results indicate that smart city construction significantly improves the health of residents, though the effect emerges with a time lag. The findings are consistently supported across robustness checks. Mechanism analysis reveals that smart city construction improves public health by raising income levels, increasing opportunities for physical exercise, and improving air quality. Further analysis shows that the health effects of smart city construction are heterogeneous across regions and city sizes. Theoretical implications and targeted policy recommendations are provided to promote public health in the context of smart city advancement.

## 1 Introduction

Rapid urbanization has given rise to pressing challenges, including resource depletion, air pollution, and escalating public health risks. As a result, the concept of the smart city has gained increasing global attention. Smart cities represent an emerging approach of social development. Although no unified definition has been established, scholars generally agree that smart city development improves resource efficiency, enhances governance, and promotes better living conditions for residents (1).

It is well established that living conditions are fundamental determinants of public health (2). Better air quality and enhanced provision of urban public services have been shown to contribute to public health gains (3). Research from the United States confirms that air pollution reduction improves health (4), with studies from African economies yielding consistent results (5). Growing scholarly attention has been devoted to examining how smart city development improves urban living conditions. The implementation of digital and communication technologies in smart cities aims to advance residents’ well-being (6, 7). Well-designed urban planning further supports physical and mental health by optimizing living environments (8). Related work has examined the health implications of emerging technologies embedded in smart city programs (9) and has shown that information and communication technologies can reduce costs and resource use while enhancing service quality (10). Nevertheless, rigorous evidence on health effects from the perspective of new infrastructure remains limited. While much of the existing research has emphasized the environmental and economic implications of smart cities at a macro level, little attention has been directed to the causal relationship between smart city construction and public health, as well as the mechanisms underlying such effects. Our study therefore evaluates the impact of smart city development, characterized by the integration of digital and communication technologies, on public health, with the aim of informing policy.

Since the beginning of the 21st century, major countries and regions worldwide have successively initiated smart city construction. Currently, over 1,000 smart cities around the world are under construction or being initiated, and this number is expected to grow at an annual rate of 20%. China has introduced a series of policies to promote smart city development since 2010. This provides an opportunity to identify the impact of new infrastructure construction on public health. Following the launch of the first batch of national smart city pilot programs by the Ministry of Housing and Urban–Rural Development in late 2012, the number of participating cities has continued to grow. In 2021, smart city development was formally incorporated in China’s 14th Five-Year Plan for National Economic and Social Development and in the Outline of Long-Term Goals for 2035. As a key part of new infrastructure, the smart city program aims to fulfill people’s aspirations for a better life through technological innovation. According to the Guiding Opinions on Promoting the Healthy Development of Smart Cities issued by China’s National Development and Reform Commission, the core objective of smart city construction is to enhance residents’ sense of well-being, with health identified as a central factor.

This paper advances existing research by exploring the impact of smart city construction on public health from a distinctive perspective of new infrastructure development, drawing on the Chinese experience. Diverging from previous studies that emphasize macro level patterns, we conduct a resident level analysis based on 18,993 observations. Treating the pilot program in China as a quasi-natural experiment, we employ a multi-period difference-in-differences approach to estimate the impact of smart city construction on public health. A comprehensive mechanism analysis is conducted to explore the channels through which the effects operate. The findings are consistently supported across robustness checks. To account for endogeneity, a reliable instrumental variable is adopted to address potential concerns. Further analysis reveals the heterogeneity of such impact. These findings not only enrich the empirical understanding of how smart city development influences public health, but also provide practical implications for policy efforts aimed at fostering healthier urban environments.

The paper is organized as follows. Section 2 develops the theoretical framework and hypotheses. Section 3 outlines the methodology. Section 4 presents the empirical results. Section 5 offers concluding remarks.

## 2 Theoretical development and hypotheses

Smart cities offer a novel approach to urban governance, relying on new infrastructure, particularly digital and intelligent technologies, to foster resilient, inclusive, and sustainable urban growth. Existing studies indicate that smart cities significantly contribute to improved quality of life and public health by optimizing service delivery and fostering resilient systems (11, 12). A critical objective of smart city construction is to achieve sustainable development (13), with access to public services and infrastructure playing a central role. Smart cities are grounded in a people-centered philosophy, addressing the diverse needs of residents while emphasizing inclusivity, equity, and civic engagement. By integrating new infrastructure into urban planning and healthcare systems, smart cities optimize the allocation of medical resources (32). The application of ICT improves the operation of urban systems, thereby facilitating more effective public health services (14).

Digital platform development drives the integration of household health data, resulting in expanded healthcare coverage (15). Meanwhile, the concept of smart health incorporates technologies into urban emergency response systems, supporting a shift from reactive to preventive care models (16). Empirical studies show that cities with robust IoT architectures demonstrate greater responsiveness to public health shocks, leading to improved individual health outcomes (17).

Beyond advancements in health service, smart city construction promotes green development through the adoption of environmentally sustainable practices. These initiatives help create health-friendly living conditions and reduce exposure to health risks. Combined with efficient, smart cities exert a positive influence on the physical and mental well-being of residents (9).

Based on the above analysis, we propose the following hypothesis:

*H1*: Smart city construction has a positive impact on public health.

Technological innovation in smart cities contributes to increased income levels among residents. The integration of ICT with sustainable urban planning creates new drivers of economic growth. Ecology-oriented urban development promotes industrial upgrading and enhances residents’ living conditions.

Smart city construction strengthens information infrastructure and supports the development of digital platforms. The establishment of new digital infrastructure fosters innovation ecosystems and increases urban attractiveness to emerging industries (18). According to Chen et al. (19), smart city development improves administrative efficiency and optimizes spatial planning, thereby reinforcing urban competitiveness. Economic vitality is further stimulated through the application of advanced technologies, which attracts business investment. Capital inflows lead to more employment and higher disposable income, ultimately contributing to better health outcomes.

Moreover, smart city development promotes inclusive economic growth by creating income opportunities for marginalized groups (19). Such inclusion increases labor force participation and drives higher average wage levels. Enhanced quality of life, in turn, leads to better health outcomes. In addition, better governance in smart cities promotes further income growth and strengthens public trust in health-related policies (20). A more livable urban environment also encourages positive lifestyle attitudes and supports overall health.

Drawing on the above arguments, we propose the second hypothesis:

*H2*: Smart city construction improves public health by increasing income levels.

Smart city construction holds considerable potential to enhance physical activity and improve overall public health outcomes. Community engagement in physical activity is shaped not only by personal characteristics but also by environmental factors, such as the accessibility of sports facilities. Smart cities support healthy lifestyles and encourages exercise-friendly urban environments. New urban infrastructure promotes participation in physical activity (21). Empirical studies have demonstrated that the development of smart city parks and leisure facilities effectively reduces barriers to exercise, thereby increasing residents’ engagement in sports activities (22). Park and Fujii (23) emphasize that people-centered urban design, when aligned with local preferences, significantly enhances residents’ willingness to utilize fitness resources and engage in physical activity.

Data-driven urban design also contributes significantly to promoting active lifestyles. Urban intelligence has integrated emerging technologies into the sports industry, leading to an increase in exercise frequency among residents. The development of digital platforms and new technology-enabled activity scenarios not only provides diverse exercise options for sports enthusiasts, but also encourages more active participation in physical activities (24).

Meanwhile, smart city construction improves personalized exercise experiences, enhancing residents’ satisfaction. Supported by integrated data platforms and digital infrastructure, residents’ physical profiles, preferences, and activity data can be continuously recorded and analyzed, promoting more scientific and tailored exercise guidance. Yue (25) finds that leveraging public health data allows urban planners to formulate targeted public policies, improve the quality of physical education, and effectively promote youth participation in regular physical activity.

Building on the preceding analysis, we formulate the third hypothesis as follows:

*H3*: Smart city construction improves public health by promoting physical exercise.

Air pollution remains one of the most pressing challenges to achieving high-quality urban development. Smart city construction inherently encompasses the principles of green and low-carbon development. By integrating emerging technologies with data-driven solutions, such programs foster innovation in urban governance and contribute to a significant reduction in air pollution. A clean atmosphere and healthy living environment are closely associated with improved health outcomes.

Empirical evidence indicates that green urban planning can significantly improve air quality by reducing atmospheric pollutants (26). Smart cities facilitate the deployment of air quality monitoring systems, enabling the effective tracking of pollution. The integration of environmental sensors into urban infrastructure allows for accurate, real-time monitoring and provides reliable data for assessing air quality. These networks can detect emerging pollution trends and issue early warnings. In addition, residents can actively participate in monitoring and data collection through mobile applications. The availability of real-time air quality information raises public awareness of pollution exposure and encourages individuals to make health-related decisions (27). Furthermore, deep learning models can predict individual exposure to harmful pollutants, thereby offering valuable support for more precise public health policies (28).

Accordingly, the following hypothesis is proposed:

*H4*: Smart city construction improves public health by improving air quality.

**Figure 1:**
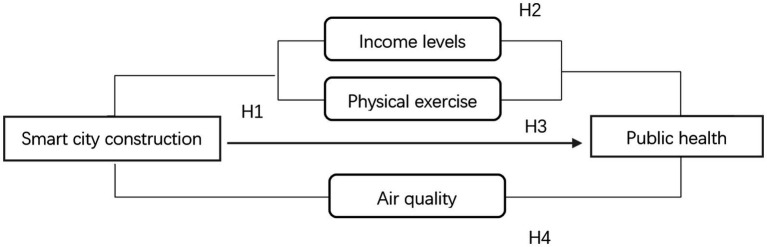
Theoretical framework. Source: by authors.

The theoretical framework is depicted in Figure 1.

## 3 Methodology

### 3.1 Specification

The construction of smart cities in China began in late 2012 with the launch of the first batch of pilot cities by the Ministry of Housing and Urban–Rural Development, followed by two successive rounds of pilot city designations. In our analysis, we treat the staggered rollout of the smart city policy as a quasi-natural experiment. Following the approach recommended by Beck et al. (29), we employ a multi-period difference-in-differences (DID) approach to estimate the impact of smart city construction on public health. Specifically, our benchmark specification is:


(1) $$\text{health}y_{\mathrm{ijt}}=a_{0}+a_{1}{\text{treat}}_{i}\cdot {\text{post}}_{t}+\beta X_{\mathrm{ijt}}+\theta W_{\mathrm{it}}+\mu_{i}+\gamma_{t}+\xi_{\mathrm{ijt}}$$

In Equation 1, *healthy_ijt_* stands for the health status of individual 𝑗 in city i at time t. *treat_i_* is a dummy variable indicating whether city i is included in the smart city pilot program. It equals 1 if city 𝑖 is included in the smart city pilot program, and 0 otherwise. *post_t_* is a time dummy equal to 1 for years following the implementation of the smart city program, and 0 otherwise. The interaction term *treat_i_·post_t_* serves as the variable “smart city construction,” with α_1_ capturing the estimated policy effect. *X_ijt_* denotes a vector of individual-level control variables, while *W_it_* represents a vector of city-level controls. *γ_t_* and *μ_i_* represents year and city fixed effects, respectively. *ξ_ijt_* is the random error term.

### 3.2 Variable definition

#### 3.2.1 Dependent variable

The core dependent variable in our study is public health *healthy*. To comprehensively capture individual health conditions, we employ a self-reported health indicator based on respondents’ subjective assessment in the CFPS survey. Respondents were asked: “How would you rate your current health status?” Responses were categorized into five levels: unhealthy, average, relatively healthy, healthy, and very healthy, coded from 1 to 5, with higher values indicating better perceived health. In the robustness analysis, we employ an objective health indicator based on the absolute deviation of an individual’s actual Body Mass Index (BMI) from an ideal benchmark. Following Duan (30), we set 22 as the ideal BMI. The objective health indicator is thus defined as the absolute value of the difference between an individual’s actual BMI and 22.

#### 3.2.2 Core independent variable

The core independent variable is the smart city construction policy. The interaction term treat_i_*post_t_ serves as the DID estimator. *treat_i_* is a binary variable equal to 1 if city 𝑖 was included in the national smart city pilot list during the period 2013–2015, and 0 otherwise. *post_t_* is a time dummy variable that equals 1 in the year following the policy implementation and in all subsequent years, and 0 otherwise.

#### 3.2.3 Intermediary variables

Three intermediary variables are used to explore the potential mechanisms through which smart city construction may influence public health.

The first intermediary variable is residents’ income level (*income*). It is measured using responses to the following CFPS survey question: “In the past 12 months, taking into account wages, bonuses, cash benefits, and in-kind subsidies, and after deducting taxes and social security contributions, how much did you earn per month on average from this job?” The logarithm of this value is used as a proxy for residents’ income level. The second intermediary variable is physical exercise(*train*). It is based on the CFPS question: “In the past year, how often did you engage in physical exercise during your spare time?” Responses are coded on a five-point scale:1 = never;2 = less than once a month or 1–3 times per month;3 = 1–2 times or 3–4 times per week;4 = about 5 times per week;5 = once or more per day. A higher score indicates greater physical activity intensity. The third intermediary variable is air quality(*PM*). It is proxied by the annual average concentration of fine particulate matter PM2.5. A higher concentration of PM2.5 reflects worse air quality and indicates a more polluted urban environment.

#### 3.2.4 Control variables

Control variables at the city level include medical and health infrastructure, total population, population density, economic development level, and urban industrial structure. At the individual level, control variables include gender, age, marital status, education level, household registration status, income, health care expenditure, physical exercise, social interaction, and Internet usage.

Definitions and descriptive statistics of all variables are provided in Table 1.

**Table 1:** Descriptive statistics.

Variable	Definition	Obs.	Mean	Std. Dev.
Healthy	Self-rated health status: 1–5, higher values indicate better perceived health.	18,993	3.143	1.175
BMI	BMI-based health status: logarithm of the absolute deviation from BMI 22, calculated as ln(|(weight/2) / (height/100)^2^–22|)	18,993	1.322	1.998
Smart city construction	Smart city pilot policy indicator: 1 = treated; 0 = not treated.	18,993	0.092	0.281
Gender	Gender: 1 = male; 0 = female.	18,993	0.533	0.489
Age	Age in years.	18,993	45.899	9.985
Marriage	Marital status: 1 = married; 0 = unmarried.	18,993	0.956	0.321
Edu	Education level: 1–8,1 = illiterate; 8 = doctoral degree	18,993	4.987	2.463
Register	Household registration status: 1 = non-agricultural; 0 = agricultural.	18,993	0.759	0.433
income	Residents’ income: natural logarithm of monthly household income.	18,993	8.254	1.087
HealPay	Health care expenditure: natural logarithm of household spending on health and fitness in the previous year.	18,993	1.067	2.58
Train	Physical exercise: 1–5, higher values indicate greater intensity.	18,993	3.251	0.835
Social	Frequency of social interaction: 1 = frequent; 0 = infrequent.	18,993	0.613	0.479
Internet	Internet usage: 1 = used; 0 = otherwise.	18,993	0.574	0.492
PM	Air quality: natural logarithm of annual mean PM2.5 concentration.	18,993	1.342	1.988
Medical	Medical condition: number of hospital beds per 1,000 residents.	1,375	5.169	1.172
Population	Urban population: natural logarithm of the total population in municipal districts.	1,375	4.872	3.323
Density	Population density: 10,000 persons/km^2^ in built-up area	1,375	15.314	0.672
PerGDP	Real per capita GDP: natural logarithm of inflation-adjusted per capita GDP.	1,375	10.542	0.636
Industry	Urban industrial structure: ratio of secondary to tertiary industry output.	1,375	1.341	2.443
Smartnet	Number of internet accounts per 100 households	1,375	0.056	0.043
SmartDX	Urban innovation`: natural logarithm of urban innovation index	1,375	2.086	3.745
Altitude	Altitude: natural logarithm of altitude variation	1,375	5.298	5.127

### 3.3 Data

Our sample period covers 2010–2020, starting in 2010 when China introduced a series of policies to promote smart city development, and ending in 2020 in order to exclude potential confounding effects of the COVID-19 pandemic. The data used in our study come from two sources. First, the micro-level data are drawn from the China Family Panel Studies (CFPS) covering the period from 2010 to 2020. The CFPS is a nationally representative longitudinal survey conducted by the Institute of Social Science Survey at Peking University. It collects rich and dynamic information on individuals, focusing on social, economic, demographic, and health-related changes in China. After excluding outliers and observations with missing values, a total of 18,993 valid individual-level samples are retained. Second, the macro-level data are obtained from the China City Statistical Yearbook (2010–2020) and the official websites of local municipal statistical bureaus. Based on these sources, we compile indicators of public service provision across 30 provinces, autonomous regions, and municipalities in China (excluding the Tibet Autonomous Region) for the period 2010–2020. These macro-level indicators are subsequently matched with micro-level data by city and year.

## 4 Results

### 4.1 Baseline regression results

We treat the smart city pilot program as a quasi-natural experiment and employs the difference-in-differences approach to identify the causal effect of smart city construction on public health. The baseline regression results are presented in Table 2. Specifically, Column (1) controls for time and city fixed effects only. Column (2) incorporates individual-level control variables, Column (3) adds city-level control variables, and Column (4) includes both sets of control variables, along with time and city fixed effects.

**Table 2:** Baseline regression results.

Variable	(1)	(2)	(3)	(4)
Smart city construction	0.407*** (0.038)	0.299*** (0.092)	0.324*** (0.086)	0.231*** (0.081)
Gender		0.458** (0.223)		0.348*** (0.107)
Age		−0.078*** (0.023)		−0.653*** (0.019)
Marriage		0.678** (0.323)		0.763*** (0.116)
Edu		0.134*** (0.023)		0.135*** (0.023)
Register		−0.005 (0.031)		−0.018 (0.032)
Income		0.984*** (0.134)		0.873*** (0.095)
Healpay		0.344*** (0.106)		0.367*** (0.113)
Train		0.082*** (0.012)		0.051*** (0.013)
Social		0.516*** (0.168)		0.058** (0.024)
Internet		0.0189** (0.009)		0.0171** (0.008)
Medical			2.308*** (0.509)	1.344*** (0.413)
Population			0.912*** (0.114)	0.556*** (0.093)
Density			−1.765*** (0.228)	−0.951*** (0.114)
Pergdp			0.877** (0.345)	0.253** (0.109)
Industry			−8.179*** (2.003)	−3.011*** (0.708)
City fixed effects	Y	Y	Y	Y
Year fixed effects	Y	Y	Y	Y
Constant	2.673***	2.655***	2.782***	2.769***
	(0.0047)	(0.0064)	(0.0149)	(0.0164)
R-squared	0.0235	0.0425	0.0859	0.0873
Observations	18,993	18,993	18,993	18,993

*p < 0.1, **p < 0.05, ***p < 0.01. Robust standard errors clustered at the regional level are reported in parentheses.

Based on the regression results presented above, several key findings emerge. First, smart city construction shows a positive effect on public health, regardless of whether additional control variables are included. Specifically, Column (4) shows that smart city construction increases residents’ self-reported health by 0.231 points, which is statistically significant at the 1% level. This result, to a certain extent, illustrates how digital and information technologies contribute to the people-centered urban development. It also offers empirical support for promoting high-quality urban growth through technological innovation. Second, the estimated effects of control variables yield further insights. After accounting for key factors, cities with larger populations tend to have healthier residents. In contrast, higher population density is associated with poorer health outcomes. These findings motivate the following heterogeneity analysis. The estimated effects of the remaining control variables are broadly consistent with theoretical expectations and are not discussed further for the sake of brevity. Overall, the findings provide strong evidence that smart city construction plays a significantly positive role in improving public health.

### 4.2 Parallel trends and placebo test

**Figure 2:**
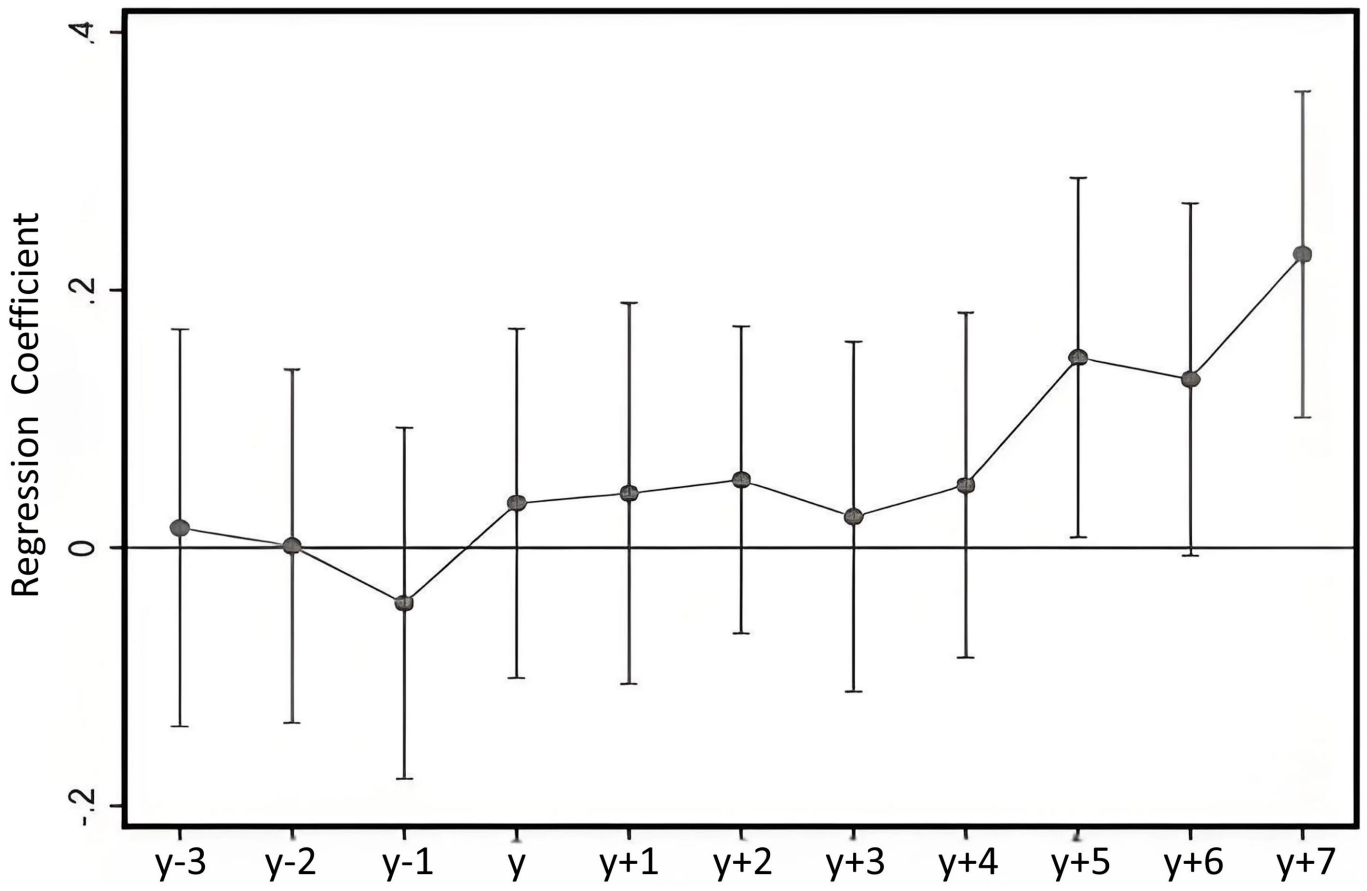
Parallel trend and dynamic treatment effects.

A key identification condition for the Difference-in-Differences approach is the parallel trends assumption, which requires that the treatment and control groups exhibit similar trends prior to the policy intervention. Figure 2 illustrates the results of the parallel trends test, using 2013 as the policy time point, along with the dynamic effects on the outcome variable over time.

As shown in Figure 2, there is no significant difference in the health trends between the treatment and control groups prior to the implementation of the smart city program, supporting the validity of the parallel trends assumption required for the Difference-in-Differences approach. After the program was implemented in 2013, the positive impact on residents’ physical health showed a generally increasing trend, although this effect emerged with a time lag. This delayed response aligns with the nature of smart city development, where the positive effects of digital infrastructure take time to materialize due to the requirement for widespread adoption.

Given that the estimated effect of smart city construction on public health may be influenced by other concurrent policy interventions, which could lead to an overestimation or underestimation of the true policy impact, we conduct a placebo test to assess the robustness of the results. Specifically, we artificially advance the policy implementation year by one (*dt-advan1*), two (*dt-advan2*) and 3 years (*dt-advan3*). Each is then interacted with the treatment group indicator to construct three interaction terms: smart city construction_advan2010, smart city construction_advan2011, and smart city construction_advan2012. These variables are subsequently included in the estimating equation. If none of the three placebo variables has a significant effect on public health, it indicates that changes in health outcomes were not influenced by any pre-policy placebo interventions. If some of the placebo variables are significant but have smaller absolute coefficients than the main policy effect (0.231), this may suggest a degree of overestimation. However, as long as the estimated effects remain in the same direction, the results can still be considered robust. Table 3 reports the results of the placebo test.

**Table 3:** Placebo test results.

Variable	(1)	(2)	(3)
Smart city construction-advan2010			0.0228**
			(0.0103)
Smart city construction-advan2011		0.0215***	
		(0.0056)	
Smart city construction-advan2012	0.0142**		
	(0.0062)		
Constant	2.5277***	2.5111***	2.5467***
	(0.0211)	(0.0204)	(0.0263)
Control variables	Y	Y	Y
City fixed effects	Y	Y	Y
Year fixed effects	Y	Y	Y
R-squared	0.4354	0.4322	0.4475
Observations	18,993	18,993	18,993

*p < 0.1, **p < 0.05, ***p < 0.01. Robust standard errors clustered at the regional level are reported in parentheses.

### 4.3 Baseline regression results

To assess the reliability of the regression results, we conduct several robustness checks.

First, we replace the dependent variable with the natural logarithm of the absolute deviation from the ideal BMI value. Column (1) of Table 4 reports the regression result using the indicator for smart city construction as the core independent variable.

**Table 4:** Robustness checks.

Variable	(1)	(2)	(3)	(4)	(5)	(6)
	Smart city construction	FE	FE	IV-2SLS	FE	IV-2SLS
Smart city construction	−0.134***					
	(0.0233)					
Smartnet		−0.115***		0.0568***		
		(0.0247)		(0.0114)		
Smartdx			0.099**		0.0811*	0.0775**
			(0.047)		(0.0416)	(0.0353)
Constant	1.080***	5.117***	1.185***		1.792***	
	(0.0991)	(0.341)	(0.0918)		(0.29)	
Control variables	Y	Y	Y	Y	Y	Y
City fixed effects	Y	Y	Y	Y	Y	Y
Year fixed effects	Y	Y	Y	Y	Y	Y
R-squared	0.2646	0.2194	0.5126	0.4451	0.5482	0.4934
F statistic				11.68 [0.0006]		15.48 [0.0001]
Kleibergen-Paap rk LM statistic				13.804 [0.0002]		12.888 [0.0016]
Kleibergen-Paap rk Wald F statistic				26.347		27.312
Observations	18,993	18,993	18,993	18,993	18,993	18,993

*p < 0.1, **p < 0.05, ***p < 0.01. Robust standard errors clustered at the regional level are reported in parentheses.

Second, we replace the core independent variable. The original interaction term *treat_i_·post_t_* is replaced with two alternative indicators reflecting urban smart development. One proxy is *smartnet*, defined as the number of internet users per 100 people, calculated from the number of internet broadband users and the total population based on data from the China City Statistical Yearbook. Another proxy is *smartdx*, the urban innovation index developed by Fudan University. Columns (2) and (3) of Table 4 display the regression results using these proxy indicators.

Third, to address potential endogeneity concerns, we further conduct an instrumental variable (IV) estimation. A valid exogenous IV must meet both the relevance and exogeneity conditions. We use terrain undulation, measured as the standard deviation of altitude within each sample area, as the instrumental variable. This decision is shaped by various considerations. Network infrastructure constitutes a core element of smart city development, and existing studies indicate that terrain undulation significantly increases the cost of infrastructure deployment (33). Generally, areas with more complex terrain and steeper elevation gradients face higher costs for network infrastructure deployment. In addition, terrain undulation also affects internet signal quality, with mountainous and hilly areas showing weaker coverage than flat regions. Thus, urban terrain variation is strongly correlated with smart city development. At the same time, terrain variation does not directly affect public health, satisfying the exogeneity condition. Therefore, using terrain undulation as an instrumental variable is appropriate. Based on the regression model in column (3), we use the natural logarithm of terrain undulation (*altitude*) as the external IV, and conduct two-stage least squares (2SLS) regression. The estimation results are reported in Column (4).

Table 4 also reports the results of IV validity tests. The F-test in the first-stage regression rejects the null hypothesis of no correlation between the instrumental variable and the endogenous core independent variables (*smartnet* and *smartdx*), indicating a strong linear relationship. The Kleibergen-Paap rk LM test yields *p*-values well below 1%, rejecting the null of underidentification and confirming that the instrument is statistically capable of identifying the endogenous variables. Given the use of robust standard errors, the Kleibergen-Paap rk Wald F statistic is employed to test for weak instruments. The *F*-values for the two models are 26.347 and 27.312, both substantially exceeding conventional threshold values, indicating that the instrument is not weak and possesses sufficient explanatory power. Accordingly, it can be concluded that the instrumental variables used are statistically valid.

From the regression results presented in Table 4, the coefficients of both *smartnet* and *smartdex* remain positive and statistically significant at the 10% level after replacing the core independent variables, reinforcing the robustness of the association between smart city development and public health. To address potential endogeneity, the study employs IV estimation using two-stage least squares (2SLS). The results are consistent with the baseline estimates in both sign and significance, supporting a positive causal effect of smart city construction on public health.

### 4.4 Mechanism analysis

To empirically test the potential mechanisms through which smart city construction affects public health, we follow the mediation effect approach proposed by Baron and Kenny (31) and specify Equations 2 and 3 as follows:


(2) $$M_{\mathrm{ijt}}=c_{0}+a\times {\text{treat}}_{i}\cdot {\text{post}}_{t}+\beta X_{\mathrm{ijt}}+\theta W_{\mathrm{it}}+\mu_{i}+\gamma_{t}+\xi_{\mathrm{ijt}}$$

(3) $$\text{health}y_{\mathrm{ijt}}=c_{0}+b\times M_{\mathrm{ijt}}+\beta X_{\mathrm{ijt}}+\theta W_{\mathrm{it}}+\mu_{i}+\gamma_{t}+\xi_{\mathrm{ijt}}$$

If both coefficients a and b are statistically significant in the above equations, it indicates that the variable “smart city construction” has a significant mediation effect on public health through the mediator *M*_*ijt*._ The mediators examined include residents’ income level (*income*), intensity of physical exercise (*train*), and air quality (*PM*). Table 5 reports the estimation results.

**Table 5:** Mechanism analysis.

Variable	(1)	(2)	(3)	(4)	(5)	(6)
	Residents’ income		Physical exercise		Air quality	
	Income	Healthy	Train	Healthy	PM	Healthy
Smart city construction	0.0513*** (0.0148)		0.0676** (0.0332)		0.0776*** (0.0211)	
Income		0.728*** (0.0608)				
Train				0.9414*** (0.1136)		
PM						0.4452* (0.2608)
Control variables	Y	Y	Y	Y	Y	Y
City fixed effects	Y	Y	Y	Y	Y	Y
Year fixed effects	Y	Y	Y	Y	Y	Y
Constant	4.7798*** (0.0141)	3.0637*** (0.0102)	3.5482*** (0.0267)	2.7678*** (0.0198)	1.7687*** (0.0302)	2.7804*** (0.025)
R-squared	0.3214	0.2739	0.1874	0.2117	0.1697	0.3459
Observations	18,993	18,993	18,993	18,993	18,993	18,993

*p < 0.1, **p < 0.05, ***p < 0.01. Robust standard errors clustered at the regional level are reported in parentheses.

Specifically, regarding the income mechanism, Columns (1) and (2) of Table 5 show that the coefficients on the smart city policy variable and residents’ income level (*income*) are both significantly positive at the 1% level. This indicates that smart city construction significantly increases residents’ income, which in turn contributes positively to their health.

As for the physical exercise mechanism, Columns (3) and (4) demonstrate that both the smart city policy variable and the exercise variable (*train*) are significantly positive at the 5% level. This suggests that smart city construction promotes higher levels of physical activity, thereby enhancing public health.

With respect to the air quality mechanism, Columns (5) and (6) reveal that the coefficients on the smart city policy variable and air quality (*PM*) are both significantly positive at the 1% level, indicating that smart city construction leads to improved air quality, which subsequently has a significant positive effect on public health.

### 4.5 Heterogeneous effects

China’s vast territory is characterized by significant spatial and regional disparities in economic development. According to the general patterns of spatial economic evolution, the eastern region enjoys the highest level of economic development, followed by the central and northeastern regions, while the western region remains relatively underdeveloped. Based on this spatial classification, we divide the sample cities into four groups: eastern, central, western, and northeastern regions. As reported in Table 6, smart city construction has a statistically significant positive effect on public health at the 5% level in the eastern and central regions. However, such a significant effect is not observed in the northeastern and western regions. Several reasons may account for this disparity. The eastern and central regions benefit from stronger economic foundations, more developed service sectors, greater openness to innovation, and more advanced urban infrastructure, all of which enhance the positive effects of smart city development. As a result, these regions experience relatively faster industrial upgrading, leading to higher resident income levels. Higher income, in turn, enables individuals to prioritize their health more effectively. Moreover, cities in the eastern and central regions demonstrate higher levels of operational efficiency and digitalization. Smart transportation systems function effectively, and urban air quality has improved considerably, contributing to better living conditions. In addition, residents in these regions have greater access to smart fitness devices and intelligent exercise facilities, which increase the flexibility of physical activity and support more convenient exercise opportunities.

**Table 6:** Heterogeneity effects by geographic region.

Variable	Eastern	Central	Western	Northeastern
Smart city construction	0.0537**	0.0338*	0.0588	0.1876
	(0.0257)	(0.0186)	(0.1765)	(1.105)
Constant	2.46***	3.57***	2.75***	3.69***
	(0.367)	(0.876)	(0.913)	(0.907)
Control variables	Y	Y	Y	Y
City fixed effects	Y	Y	Y	Y
Year fixed effects	Y	Y	Y	Y
R-squared	0.2985	0.2756	0.1935	0.2490
Observations	6,899	4,167	5,692	2,235

*p < 0.1, **p < 0.05, ***p < 0.01. Robust standard errors clustered at the regional level are reported in parentheses.

City population may also influence public health. Following the 2014 classification standard for urban population in China, cities are grouped by population size. Table 7 presents the subgroup regression results. The results reveal heterogeneous effects of smart city construction across cities, depending on their population size. Specifically, among large cities with populations exceeding 1 million, the positive effect of smart city construction on public health becomes more pronounced as population size increases. In contrast, the effect is not statistically significant in small and medium-sized cities with populations below 1 million. A possible explanation is that larger cities adopt smart and digital infrastructure more rapidly. Accelerated upgrades in urban management and technology foster economic development, improve quality of life, and increase residents’ access to physical exercise opportunities.

**Table 7:** Heterogeneity effects by population.

Variable	pop∈ (0, 50)	pop∈ (50, 100)	pop∈ (100, 300)	pop∈ (300, +∞)
Smart city construction	0.0684	0.0834	0.0639*	0.0899**
	(0.1248)	(0.1029)	(0.0345)	(0.0434)
Constant	3.143***	2.844***	2.505***	2.972***
	(0.041)	(0.025)	(0.027)	(0.079)
Control variables	Y	Y	Y	Y
City fixed effects	Y	Y	Y	Y
Year fixed effects	Y	Y	Y	Y
R-squared	0.2116	0.3317	0.3124	0.2713
Observations	3,674	3,788	4,632	8,677

*p < 0.1, **p < 0.05, ***p < 0.01. Robust standard errors clustered at the regional level are reported in parentheses.

## 5 Conclusion

### 5.1 Theoretical implications

This paper offers new evidence regarding the impact of smart city initiatives on public health by situating the analysis within the broader framework of new infrastructure development. Exploiting the staggered rollout of smart city pilot program in China as a quasi-natural experiment, the study applies a multi-period DID approach to obtain credible causal estimates. It further investigates the underlying mechanisms through a systematic analysis of channels. Robustness checks corroborate the findings, while heterogeneity analysis reveals differentiated effects across groups.

The main findings of this paper are as follows. First, the construction of smart cities has a significantly positive impact on public health. Second, smart city development improves physical health outcomes through multiple channels, including increasing household income, enhancing opportunities for physical exercise, and improving air quality. Third, the health effects of smart city construction are heterogeneous across regions and city sizes. Specifically, the positive impact is statistically significant at the 5% level in the eastern and central regions, but not in the northeastern and western regions. In terms of city size, the positive effect is more pronounced in large cities with populations exceeding 1 million, whereas no significant effect is observed in small and medium-sized cities. Thus, this study advanced the understanding of unban development on public health, thereby advocating for a city design.

### 5.2 Practical implications

These findings carry several policy recommendations. First, smart city initiatives should be prioritized as a strategic means to improve public health. Beyond investing in robust ICT backbones, governments should actively drive the integration of digital technologies across urban governance, public healthcare, and public services. Integrating AI-enabled screening and remote monitoring across connected care systems, while linking these tools to public health data, helps to expedite clinical responses. Public health platforms might integrate IoT surveillance, while public fitness facilities are recommended to be upgraded to encourage physical activity among residents.

Second, promote the coordinated integration of smart city development with urban development strategies. Smart city programs grounded in new infrastructure should be advanced with a clear focus on improving public health. Healthy community planning should incorporate intelligent facilities and environmental monitoring systems into neighborhood design. Meanwhile, projects in smart healthcare, intelligent aging care, and related domains should be supported, with demonstration pilots rolled out into routine practice. Additionally, broaden digital financial inclusion so that all population groups can access and benefit from new infrastructure services, advancing smart city development and public health concurrently.

Third, a nationally coordinated smart city strategy is encouraged to mitigate spatial inequality and ensure equitable access to the benefits of digital urban transformation. Smart city development should extend beyond large metropolitan areas. Despite relatively weaker foundations, small and medium-sized cities play a crucial role in national urbanization. Efforts must be made to prevent regional divergence by extending successful models from more developed to less-developed regions. Financial support, targeted technical training, and cross regional knowledge-sharing platforms would help ensure regionally balanced development of digital capacities that support public health. Locally adapted design should be tailored to ensure the practicality of new infrastructure and smart services, contributing to a more balanced and inclusive pattern of urban development.

## Data Availability

Publicly available datasets were analyzed in this study. This data can be found at: The micro-level data are from the China Family Panel Studies (CFPS), administered by the Institute of Social Science Survey (ISSS) at Peking University and accessible at: http://www.isss.pku.edu.cn/cfps/. The macro-level data were obtained from the China Urban Statistical Yearbook, published by the National Bureau of Statistics of China and available at: https://www.stats.gov.cn/, as well as from the official websites of various municipal statistics bureaus. These data are publicly available.
